# Gestation-suppressed serum TSH levels during early pregnancy are not associated with altered maternal and neonatal outcomes

**DOI:** 10.1530/ETJ-23-0112

**Published:** 2023-10-18

**Authors:** Emna Jelloul, Georgiana Sitoris, Flora Veltri, Pierre Kleynen, Serge Rozenberg, Kris G Poppe

**Affiliations:** 1 Endocrine Unit Centre Hospitalier Universitaire (CHU) Saint Pierre, Université Libre de Bruxelles (ULB), Rue Haute, Brussels, Belgium; 2 Departement of Gynecology and Obstetrics, Centre Hospitalier Universitaire Saint Pierre, Université Libre de Bruxelles (ULB), Rue Haute, Brussels, Belgium

**Keywords:** pregnancy, serum TSH, pregnancy outcomes

## Abstract

**Objective:**

The aim of the study was to investigate the impact of suppressed serum TSH levels (sTSH) during early pregnancy on maternal and neonatal outcomes.

**Methods:**

In this single-centre, retrospective cohort study 1081 women were screened at 11.8 ± 2.4 weeks of pregnancy for TSH, free T4 (FT4) and TPOAb. Exclusion criteria were twin- and assisted- reproduction pregnancies, women with TSH levels >3.74 mIU/L, severe hyperthyroidism, treated for thyroid dysfunction before or after screening and gestational blood sampling <6 or >16 weeks of pregnancy. The prevalence of adverse pregnancy outcomes was compared between the study group sTSH (TSH: < 0.06 mIU/L; *n* = 36) and euthyroid controls (TSH: 0.06–3.74 mIU/L;* n* = 1045), and the impact of sTSH on pregnancy outcomes verified in logistic regression analyses.

**Results:**

Median (IQR) serum TSH level in women with sTSH was 0.03 (0.03–0.03) vs 1.25 (0.81–1.82) mIU/L in controls and FT4 levels 18.0 (14.4–20.3) vs 14.2 (12.9–15.4) pmol/L; both *P* < 0.001. None of the women with sTSH had thyrotropin receptor antibodies. Compared with controls, the prevalence of TPOAb positivity (TAI) was comparable between groups (5.6% vs 6.6%; *P* = 0.803). The prevalence of maternal and neonatal pregnancy outcomes was comparable between the study and control group. The logistic regression analyses with corrections for TAI, FT4 and demographic parameters confirmed the absence of an association between sTSH, and the following outcomes: iron deficient anaemia (aORs (95% CI)): 1.41 (0.64-2.99); *P* = 0.385, gestational diabetes: 1.19 (0.44–2.88); *P* = 0.713, preterm birth: 1.57 (0.23–6.22);*P* = 0.574 and low Apgar-1′ score: 0.71 (0.11–2.67); *P* = 0.657.

**Conclusions:**

Suppressed serum TSH levels during the first to early second trimester of pregnancy were not associated with altered maternal or neonatal outcomes.

## Introduction

The guidelines on the management of thyroid disorders in pregnancy (ATA-GL), mention the following: ‘it is important to note that subclinical hyperthyroidism (SH) has not been associated with adverse pregnancy outcomes. Therefore, a maternal TSH concentration that is low but detectable is likely not clinically significant’ (1, 2). However, no specific recommendation for or against repeat thyroid function testing was made and nothing was mentioned about suppressed TSH levels (sTSH) (1, 2). Since the ATA publication in 2017, other studies have associated SH with an increased prevalence of preterm delivery (PTB) (3, 4, 5), pre-eclampsia (6, 7) and low birth weight (3). On the contrary, a meta-analysis did not associate SH with a low-birth weight (8).

Discrepant results between recent studies and those published before the ATA-GL might be due to heterogeneity in used methodology (TSH included as a continuous or fully suppressed value), study including a larger number of women (by the use of individual-participant data meta-analysis), the inclusion of women with different backgrounds (and thus different iodine status and BMI levels), and adjustments made for other variables (hCG and iron reserve) (3, 7, 9).

Therefore, the aim of this study was to investigate the impact of sTSH levels during the late first to early second trimester on different maternal and neonatal outcomes (iron deficient anaemia (IDA), gestational diabetes (GDM), PTB and low Apgar score at 1’) using logistic regression analyses with adjustments for other thyroid, demographic and obstetric parameters.

## Materials and methods

### Overall study design/definitions

The obstetric clinic is part of the public university hospital CHU Saint Pierre in Brussels, Belgium. In this retrospective cohort study, we included women with ongoing pregnancies, who performed their biochemical work-up (including the oral glucose tolerance test (OGTT)) and obstetric follow-up entirely in our centre, during the period 2 January 2013 to 31 December 2014.

During the first antenatal consultation, demographic parameters and obstetric data were encoded and systematically completed with a biochemical analysis, including serum TSH, FT4, thyroid peroxidase antibodies (TPOAb), ferritin measurement, and in a later stage of pregnancy an OGTT.

Exclusion criteria for this study were women with multiple and assisted (ART) pregnancies, known diabetes mellitus, thyroid disorders before pregnancy (overt hyperthyroidism (FT4 levels: >30 pmol/L) and TSH levels: >3.74 mIU/L), and women treated with LT4 or antithyroid drugs before/after screening.

To improve the accuracy of ascertainment of GDM, we included only women who performed an OGTT and therefore, women with fasting glycaemia levels ≥92 mg/dL during the initial screening and/or a first trimester miscarriage were not included in the study (introducing a certain bias). Finally, we matched cases and controls for the gestational age at blood sampling (period 6–16 weeks with the presumed highest hCG levels).

**Figure 1:**
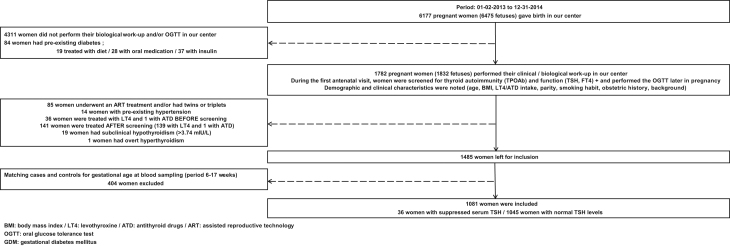
The study selection process in more detail.

In Fig. 1, we illustrate the study selection process in more detail.

Previously, we determined institutional trimester-specific limits for serum TSH and FT4 (10). Our reference range for serum TSH (2.5–97.5th percentile) during the period 11 (9–14) weeks was 0.06–3.74 mIU/L, and 10.29–18.02 pmol/L for serum FT4 (10). Thyroid autoimmunity (TAI) was defined as TPOAb levels ≥60 kIU/L. For this study, two groups were established based on TSH levels; one study group (TSH levels: <0.06 mIU/L; *n* = 36) and one control group (TSH levels between 0.06 and 3.74 mIU/L; *n* = 1045). The prevalence of baseline characteristics and pregnancy outcomes was compared between both groups.

Gestational age was based on ultrasound findings and expressed in full weeks and days of amenorrhoea. Smoking was stratified as yes/no (women who stopped smoking during pregnancy were also considered as smokers).

Outcome measures: pre-eclampsia was defined as a systolic blood pressure ≥140 mmHg and/or a diastolic blood pressure ≥90 mmHg, associated with a proteinuria >0.3 g/24 h after 20 weeks of amenorrhoea (11). Intrauterine growth restriction (IUGR) was defined as the estimated fetal weight <10th percentile for gestational age and sex.

IDA was present when haemoglobin levels are <12, 11 or 10.5 g/dL during the first, second or third trimester, respectively, together with a serum ferritin <15 µg/L. Postpartum haemorrhage was considered as blood loss ≥500 mL, and only considered after spontaneous birth. PTB was defined as birth <37 weeks. GDM was diagnosed after administration of 75 g glucose during an OGTT performed between 24 and28 weeks of pregnancy, when fasting glucose ≥92 mg/dL or 1-h postprandial glycaemia ≥180 mg/dL or 2 h ≥153 mg/dL) (12).

### Serum assay

All provisions were implemented by the laboratory of hormonology of our institution.

Serum TSH, FT4 and TPOAb levels were measured using the Chemiluminescence Centaur XP Siemens immunoanalyser. The (non-pregnant) reference values were 0.3–4.0 mIU/L, 10.3–25.7 pmol/L (0.8–2.0 ng/dL) and <60 kIU/L for TSH, FT4 and TPOAb, respectively. The total imprecision CVs were 6.9%, 4.2% and 7.6% for TSH, FT4 and TPOAb, respectively.

Serum ferritin levels were measured using the chemiluminescence Centaur XP Siemens immunoanalyser. The reference values: 15–300 µg/L and total imprecision CVs was 3.7%. Plasma glucose was measured by an automated colorimetric–enzymatic method on a Hitachi/Roche Modular P analyser; CV is 1%.

### Statistical analysis

Data were stored in a Microsoft Excel database and statistical analyses performed with StatPlus:mac, Analyst Soft Inc., statistical analysis program for macOS; version v8, and with GraphPad. Descriptive statistics are presented as mean ± s.d. for normally distributed and median (interquartile range (IQR)) for skewed variables. Comparison between the study and control group was done by *χ* ^2^ or Fisher’s exact tests (according to the number of events) for categorical data and by a *t*-test or Mann–Whitney *U* test for continuous data (according to the distribution). For the logistic regression analyses, we investigated the impact of suppressed serum TSH levels on maternal and neonatal outcomes (IDA, GDM, PTB, and low Apgar-1′). According to the outcome, corrections were made for demographic/baseline characteristics (age >30 years, obesity (BMI ≥30 kg/m²), tobacco use, and obstetric/pregnancy data (parity in the whole range, iron deficiency (ferritin levels (<15 µg/L)) and fetal gender) and thyroid parameters (FT4 in the whole range and TAI). To evaluate the discrimination ability of the model, AUC was used, to test for multicollinearity, variance inflation factors (VIFs) and for the goodness of fit of the model, the Hosmer–Lemeshow test. Results were given as adjusted odds ratios (aOR (95% CI)), and statistical tests were considered significant whenever *P* < 0.05.

## Results

Table 1 shows demographic and obstetric parameters in all women and according to the TSH group.

**Table 1:** Demographic and obstetric parameters in all women and according to TSH levels. Continuous data are expressed as mean ± s.d. or median (IQR) and categorical data as *n* (%).

	All women	Cases	Control	*P*
*n*	1081	36 (3.3%)	1045 (96.7%)	
TSH, mIU/L	<0.006–3.74	<0.06^b^	0.06–3.74^a^	
Maternal age, years	30.1 ± 5.7	30.3 ± 5.5	30.1 ± 5.7	0.865
Maternal age >30 years	505 (46.7%)	14 (38.9%)	491 (47.0%)	0.338
Pre-pregnancy				
BMI (kg/m²)	25 (22–28)	24 (22–28)	25 (22–28)	0.477
Obesity (BMI ≥30 kg/m²)	193 (17.9%)	8 (22.2%)	185 (17.7%)	0.486
Multiparity (>2)	133 (12.2%)	6 (16.7%)	127 (12.2%)	0.418
History of ≥2 first trimester MC	78 (7.2%)	3 (8.3%)	75 (7.2%)	0.792
Smoking during pregnancy	156 (14.4%)	2 (5.6%)	154 (14.7%)	0.123
Ferritin levels (µg/L)	23 (13–43)	19 (12–40)	23 (13–43)	0.301
Iron deficiency (ferritin <15 µg/L)	315 (29.1%)	15 (41.7%)	300 (28.7%)	0.093
Fetal gender (% female)	557 (51.5%)	22 (61.1%)	535 (51.2%)	0.242

^a^Normal TSH; ^b^Suppressed TSH.

MC, miscarriage.

Mean age of all women was 30.1 ± 5.7 years and median (Q1–Q3) BMI was 25 (22–28) kg/m^2^, comparable between both groups (*P* = 0.865 and 0.477). The percentage of smoking, multiparity, history of ≥2 miscarriages, iron deficiency and female fetal gender were comparable between groups.

Table 2 shows thyroid parameters in all women and according to the TSH group.

**Table 2:** Thyroid parameters in all women and according to TSH levels. Continuous data are expressed as median (IQR).

	All women	Cases^a^	Controls^b^	*P*
Gestational age (weeks)^c^	11.8 ± 2.4	11.4 ± 2.4	11.8 ± 2.4	0.298
TSH (mIU/L)	1.20 (0.74–1.75)	0.03 (0.03–0.03)	1.25 (0.81–1.82)	**<0.001**
FT4 (pmol/L)	14.2 (12.9–15.4)	18.0 (15.4–20.3)	14.2 (12.9–15.4)	**<0.001**
TPOAb (kIU/L)	28 (28–37)	28 (28–32)	28 (28–37)	0.129
TAI (TPOAb ≥60 kIU/L)	71 (6.6%)	2 (5.6%)	69 (6.6%)	0.803

^a^Women with suppressed TSH; ^b^Women with normal TSH; ^c^Gestational age at blood sampling.

FT4, free thyroxine; TAI, thyroid autoimmunity; TPOAb, thyroid peroxidase autoantibodies.

Gestational age at blood sample for all women, cases and controls was 11.8 ± 2.4 weeks. Due to the matching between cases and controls, levels were comparable between both groups (*P* = 0.298).

Median serum TSH for all women was 1.20 (0.74–1.75) mIU/L; 0.03 (0.03–0.03) mIU/L in the study group vs 1.25 (0.81–1.82) mIU/L in the control group; *P* < 0.001. The median serum FT4 for all women was 14.2 (12.9–15.4) pmol/L and in the study group was 18.0 (15.4–20.3) pmol//L vs 14.2 (12.9–15.4 pmol/L) in the control group; (*P* < 0.001). The prevalence of TAI was 6.6% for all women without differences between the study and control group (5.6 vs 6.6%; *P* = 0.803).

Table 3 shows the maternal and neonatal pregnancy outcomes in all women and according to the TSH group.

**Table 3:** Maternal and neonatal pregnancy outcomes in all women and according to TSH levels. Continuous data are expressed as median (IQR) and categorical data as *n* (%).

	All women	Cases^a^	Controls^b^	*P*
Pre-eclampsia	45 (4.2%)	0 (0.0%)	45 (4.3%)	0.397
Iron deficiency anaemia	344 (31.8%)	12 (33.3%)	332 (31.8%)	0.843
GDM	216 (20.0%)	7 (19.4%)	209 (20.0%)	0.935
Emergency C-section	68 (6.3%)	2 (5.6%)	66 (6.3%)	0.853
Blood loss at birth ≥500 mL^c^	245 (22.7%)	9 (25.0%)	236 (22.6%)	0.594
IUGR	28 (2.6%)	0 (0.0%)	28 (2.7%)	1.000
Gestational age at birth (weeks)	39.6 (38.6–40.4)	40.1 (39.1–40.4)	39.6 (38.6–40.4)	0.329
Preterm birth	51 (4.7%)	2 (5.6%)	49 (4.7%)	0.810
Birth weight (kg)	3.3 ± 0.5	3.4 ± 0.4	3.3 ± 0.5	0.456
Macrosomia	69 (6.4%)	1 (2.8%)	68 (6.5%)	0.368
Neonatal malformation	30 (2.8%)	1 (2.8%)	29 (2.8%)	0.999
Head circumference	34.4 (33.5–35.0)	34.8 (33.6–35.5)	34.1 (33.5–35.0)	0.331
APGAR-1′ (<7)	92 (8.5%)	2 (5.6%)	90 (8.6%)	0.518
NICU admission	7 (0.7%)	0 (0.0%)	7 (0.7%)	1.000

^a^Women with suppressed TSH; ^b^Women with normal TSH; ^c^Calculated for natural births.

GDM, gestational diabetes mellitus; IUGR, intra-uterine growth restriction; NICU, neonatal intensive care unit.

For all women, the prevalence of major pregnancy outcomes was 4.2% for PE, IDA 31.8%, GDM 20% preterm birth 4.7%, macrosomia 6.4% and low Apgar-1′ score 8.5% without differences between the study and control group. Other results not shown in the tables. Of the 36 women with sTSH, 23 had a SH (63.9%) and 13 overt hyperthyroidism (36.1%).

In 33 of the 36 women (91.7%) a second TSH measurement was available (initially at 11.3 ± 2.15 weeks versus 18.9 ± 4.3 for the control) and from them, 29 (87.9%) normalised their TSH levels. In 19 of the 36 women (52.8%), thyrotropin receptor antibodies (TSH-R-Ab) were measured and negative (including the four women with an sTSH at control). Two women of the 36 had positive TPOAb levels (5.6%); one with a persistent sTSH at the second measurement (but no TSH-R-Ab), and one who normalised her serum TSH (but no data on TSH-R-Ab available).

The results of the logistic regression analyses after adjustment for FT4, TAI, parity, smoking, iron deficiency and the fetal gender showed no significant association between sTSH levels (<0.06 mIU/L) and outcome IDA (aORs (95% CI)): 1.41 (0.64–2.99), *P* = 0.385; GDM: 1.19 (0.44–2.88), *P* = 0.713; PTB: 1.57 (0.23–6.22), *P* = 0.574 and low Apgar-1′ score: 0.71 (0.11–2.67), *P* = 0.657.

The model had a poor discrimination ability (AUC between 0.57 and 0.66, depending on the investigated outcome). No issues with collinearity were noted (VIFs <4), and all models passed the Hosmer–Lemeshow test for goodness of fit (*P*-values between 0.08 and 0.22).

## Discussion

Our study does not support an impact of sTSH (with mainly cases of SH) on several maternal and neonatal pregnancy outcomes. Studies published after the publication of the 2017 ATA-GL report contradictory results. On one hand, there were meta-analyses that did not report an impact on IUGR and LBW (8, 13, 14), and on the other hand, original studies and meta-analyses reporting a higher prevalence of PTB (3, 5), pre-eclampsia (7), placental abruption and even a lower risk of abortion (6). These more recent studies also included women with sTSH levels (and thus not only with low TSH) and considered demographic and obstetric confounders. Many of these studies were performed in China and compared to those performed in the EU or USA, East Asian women may have a higher prevalence of sTSH/SH (15), due to differences in BMI levels, food habits (iodine intake), the prevalence of TAI and other variables that might influence TSH levels (9). On the other hand, newer methods for meta-analyses using individual-participant data, allow to analyse TSH values as a continuous variable in the lower range and not only as a cut-off for SH (7). In the meta-analysis by Toloza *et al.*, lower (and higher) TSH levels were associated with a higher prevalence of pre-eclampsia, what was not the case when a TSH cut-off was applied (5). In the study by Nazarpour *et al.* the association between sTSH (<0.3 mIU/L) and PTB was only present in case of urinary iodine levels ≥150 μg/l (5). The precise underlying mechanism for that observation remains unclear, but different food habits and other demographic characteristics in Iranian women could have contributed to their study results.

Taking all studies into account, published before and after the ATA-GL, evidence is now stronger that sTSH might have an impact on certain pregnancy outcomes, but heterogeneity remains present due to differences in the methodology and/or the cause of sTSH/SH (Graves’ disease (GD) versus gestational hyperthyroidism (GTT)) and because thyroid medication usage was not always clearly mentioned as an exclusion criterion (5, 6, 7, 16). Only in one study, pregnancy outcomes were compared between women with GD versus GTT during early pregnancy (16). Definitions were a TSH <0.14 mIU/L, FT4 >20.71 pmol/L, with or without TSH-R-Ab for GD and GTT, respectively. GTT was (weakly) associated with GDM, and GD strongly with miscarriage, PTB and gestational hypertension (16). The contrasting results between our study and the current guideline recommendations might be due to a higher incidence of GDM in the Chinese population, the degree of GTT that was different compared to the only study included in the ATA-GL and finally, should it be mentioned that they did not adjust their results for confounders (but reported only relative risk) (1, 2, 16). Another point to be considered in women with sTSH during pregnancy is the level of FT4 to distinguish SH from overt hyperthyroidism. Few studies clearly distinguished their impact on pregnancy outcomes; in one study, SH was associated with higher risk of preeclampsia (OR: 5.14, 95% CI:  1.46–18.08; *P* = 0.011) and placental abruption (OR: 4.68, 95% CI: 1.02–21.51; *P* = 0.048), and overt hyperthyroidism increased the incidence of placenta previa (OR: 4.19, 95% CI: 1.22–14.38;  *P* = 0.023) (6). TAI was significantly higher in women with overt hyperthyroidism vs SH (25.6% vs 14.2%). In our study, sTSH was not associated with altered pregnancy outcomes, if we corrected for SH or overt hyperthyroidism instead of FT4 as a continuous variable in the pregnancy specific-range (data not shown). In three studies from the Consortium on Thyroid and Pregnancy, both SH and overt hyperthyroidism were not associated with PTB, birth weight and gestational hypertension, respectively (7, 14, 17).

Therefore, and to understand better the association between sTSH and pregnancy outcomes, results should always be corrected for other thyroid (TAI, FT4, FT3?) and demographic/pregnancy-related parameters, (age, BMI, serum hCG, iron and iodine levels and fetal gender). Corrections for FT4 should be made because the inverse log/linear relationship between TSH and FT4 has been challenged during pregnancy and since FT4 levels will be high/increased in women with GTT and in GD (16, 18). Adjusting for thyroid autoimmune parameters should be considered for several reasons; it may impede a normal hCG stimulation effect during the first trimester, and it is associated with impaired pregnancy outcomes as such (19, 20). Since in our study, only two women with sTSH had TAI, we were not able to notice a particular pattern in them compared with the 34 women with sTSH and no TAI. Adjusting for iron deficiency is rarely done, despite its association with an increased prevalence of PTB, fetus small for gestational age, perinatal death, and a higher prevalence of TAI (21, 22, 23). Another parameter for which in more recent studies an adjustment is made is the fetal gender. Previous studies have shown that maternal TSH levels were lower in women pregnant with a female fetus, probably due to higher hCG levels and/or other placental substances like the vascular endothelial growth factor with an impact on thyroid vascularisation (24). Moreover, in one study, thyroid disorders were associated with PTB only in case of pregnancies with a female fetus (25).

In daily practice, it remains challenging to propose recommendations for pregnant women with a sTSH. If women are treated with LT4, the dosage should be decreased to aim for a measurable serum TSH levels and a FT4 in the middle-normal range (except in case of a history of a severe thyroid cancer stage). Maraka *et al.* reported that treatment with LT4 in women with thyroid function between 2.5 and 4.0 mIU/L was associated with a higher prevalence of pre-eclampsia (26). An initial step could be to verify serum TSH a few weeks after the first measurement to exclude a GTT. Many women will normalise their TSH levels without any treatment (in our study 89%). It is noteworthy that in the current ATA-GL, no specific recommendation for or against thyroid function follow-up was made (1, 2). In case of a confirmed endogenous sTSH, treatment may be necessary on a case-by-case basis for women with GD, a toxic nodule/goitre and maybe temporally in case of severe GTT since the latter has recently also been associated with a higher prevalence of GDM (16). However, to date there is no proof that such an approach would improve pregnancy outcomes.

Other factors that might be considered in the risk stratification are previous pregnancies, demographic parameters (age and BMI), iodine, iron and hCG levels, fetal gender, pre-existing hypertension, diabetes and markers of pre-eclampsia (27).

Limitations of our study are the retrospective design, the low number of women with sTSH and cases with pre-eclampsia (due to the exclusion of women with pre-existing hypertension); the absence of TgAb, and serum hCG levels. Data on the iodine status were missing, but from a previous study performed in pregnant women in Brussels, we know there is a moderate deficiency, but without thyroid dysfunction (28). Not in all women with sTSH, TSH-R-Ab were measured (but they were in all women with a second sTSH). Finally, it should be noted that our logistic regression model had a poor discrimination ability, although it had a correct goodness of fit.

In conclusion, we did not observe altered maternal or neonatal pregnancy outcomes in women with suppressed serum TSH levels during the late first to early second trimester of pregnancy, when results were adjusted for TAI, FT4, demographic and obstetric confounders.

## Declaration of interest

K G Poppe received lecture fees from the Berlin-Chemie AG and Merck company. All authors declare that there is no conflict of interest that could be perceived as prejudicing the impartiality of the research reported. K G Poppe is on the editorial board of *European Thyroid Journal*. K G Poppe was not involved in the review or editorial process for this paper, on which he is listed as an author.

## Funding

No specific grant/fellowship from any funding agency in the public, commercial or not-for-profit sector.

## Statement of ethics

The study was performed in accordance with the guidelines of the Helsinki Declaration. The study was approved by the institutional review board ‘Comité Local d'Éthique Hospitalier, N° d'agréation: O.M. 007 AK/15-11-114/4568’, Centre Hospitalier Universitaire C.H.U. Saint Pierre, Rue Haute 322, 1000 Bruxelles. No written consent was obtained from the participants, since the study was a retrospective analysis of collected data.

## Data availability statement

Data are available for insight at request of the editor.

## Author contribution statement

EJ and GS drafted the first version of the manuscript. FV collected data and revised the manuscript. PK revised the manuscript. SR revised the manuscript and approved the final version. KGP designed and performed the study, acquired and analysed the data, revised. The manuscript and approved the final version.
